# Successful repair of spontaneous indirect bilateral carotid-cavernous fistula with coil embolization

**DOI:** 10.1093/jscr/rjab140

**Published:** 2021-04-22

**Authors:** Suzie A Gasparian, K V Chalam

**Affiliations:** Department of Ophthalmology, Loma Linda University School of Medicine, Loma Linda, CA, USA; Department of Ophthalmology, Loma Linda University School of Medicine, Loma Linda, CA, USA

## Abstract

Bilateral carotid-cavernous fistula (CCF) is a rare disease process, which portends poor visual outcome with delayed diagnosis and treatment. An 82-year-old woman presented with sudden onset of proptosis and decreased vision. A complete ophthalmologic examination along with magnetic resonance (MR) imaging of the brain and orbits, and MR angiography and venography of the brain confirmed the diagnosis of bilateral CCF. Diagnostic cerebral angiogram with concurrent coil embolization of the right cavernous sinus via left superior ophthalmic vein approach was performed. Bilateral indirect CCFs (type D CCF on the right and a type B CCF on the left) regressed completely after unilateral coil embolization. Visual acuity and limitation in extraocular movements significantly improved with complete resolution. In summary, we describe successful management of bilateral concurrent CCF with image-guided embolization and immediate recovery of vision and resolution of ophthalmological symptoms including proptosis and diplopia.

## INTRODUCTION

A carotid-cavernous fistula (CCF) is the result of an abnormal vascular communication between the carotid arterial system [the internal carotid artery (ICA) and/or external carotid artery (ECA)], and the venous channels of the cavernous sinus. They may be direct or indirect based on angiographic architecture, traumatic or spontaneous based on its etiology and stable or progressive based on hemodynamics [4, 5, 7].

Indirect CCFs are rare (30% of all CCFs) and typically result from ICA aneurysm rupture. Hypertension, collagen disorder, female gender and older age are known risk factors [1, 6]. The Barrow classification system for CCFs sub-divides them into direct (type A; direct connection between the ICA and cavernous sinus) and indirect (types B, C, D) CCFs. Type B CCF is a dural shunt between meningeal branches of the ICA and cavernous sinus (1%). Type C CCF is a dural shunt between meningeal branches of the ECA and cavernous sinus, and type D CCF is the result of fistulization between both the ICA and ECA and cavernous sinus [1].

Bilateral CCFs are rare and seen in 1–2% of patients with CCFs [1]. Spontaneous indirect bilateral CCFs of different types are rarer. We report a case of bilateral concurrent (types D and B) non-traumatic indirect CCFs successfully treated with endovascular coil embolization.

## CASE REPORT

**Figure 1:**
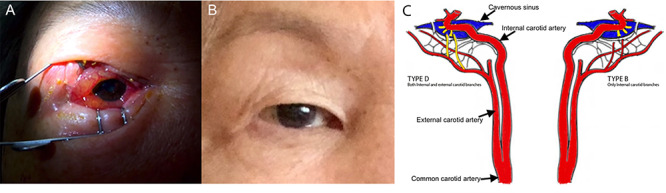
External photos of the patient’s eye before and after management of carotid-cavernous fistulas. **(A)** External photo of patient’s right eye upon presentation, prior to diagnosis of CCF. **(B)** External photos of patient’s eyes 6 months after resolution of CCFs. **(C)** Diagrammatic illustration of type D CCF on the right and type B CCF on the left as demonstrated by abnormal, yellow vasculature. Type D CCF on the right illustrates dural shunts between meningeal branches of both the internal and external carotid arteries and the cavernous sinus. Type B CCF illustrates dural shunts between meningeal branches of the internal carotid artery and cavernous sinus.

An 82-year-old female with history of macular degeneration and left frontotemporal craniotomy for meningioma resection (5 years earlier) presented with sudden onset of worsening right eye pain, proptosis, diplopia, decreased vision, and associated periorbital edema and erythema (Fig. 1).

**Figure 2:**
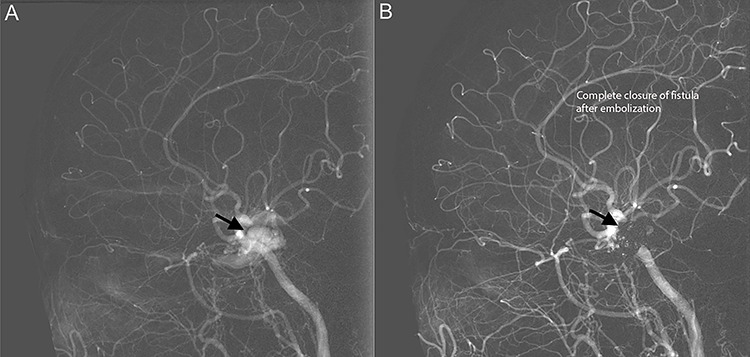
Diagnostic cerebral angiogram demonstrative of right indirect CCF. **(A)** Right ICA angiogram revealed indirect fistulization between the ECA branches and cavernous sinus (type D CCF) **(B)** with subsequent successful endovascular coil embolization.

Ocular examination revealed significant periorbital edema and erythema, moderate proptosis of the right eye. Best-corrected visual acuity (BCVA) was 20/200 in the right eye and 20/40 in the left eye. Intraocular pressure (IOP) was 33 mmHg in the right eye and 21 mmHg in the left eye. Pupillary examination revealed a sluggish, poorly reactive 3 mm pupil on the right and a reactive 3 mm pupil on the left. Extraocular movements were full in the left eye; however, there was complete ophthalmoplegia of the right eye. Anterior segment examination revealed conjunctival vascular congestion, significant chemosis and corkscrew vessels in the right eye (Fig. 1A); the left eye demonstrated mild chemosis with conjunctival corkscrew vessels prominent medially. There was no optic disk edema or choroidal folds on fundoscopy.

A computerized tomography (CT) scan of the orbits demonstrated right periorbital tissue swelling and superior ophthalmic vein thrombosis (SOVT). Magnetic resonance venography/magnetic resonance angiography (MRA) of the brain demonstrated prominent superior ophthalmic veins bilaterally with flow void extending from the septal region to the posterior orbit on the right consistent with thrombosis.

**Figure 3:**
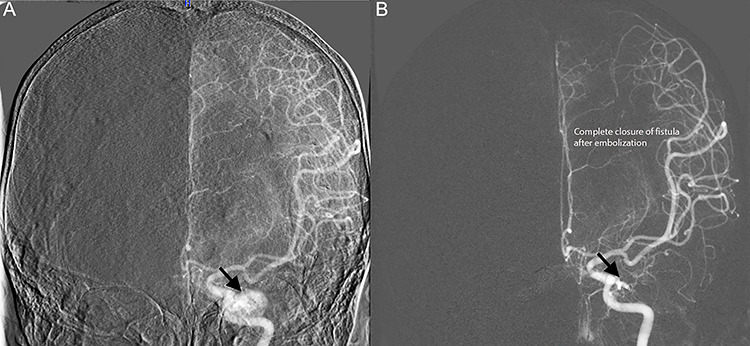
Diagnostic cerebral angiogram demonstrative of left indirect CCF. **(A)** Left ICA angiogram revealed a type B CCF supplied by branches of the meningohypophyseal trunk with inferior venous drainage **(B)** with evidence of subsequent spontaneous resolution.

Diagnostic cerebral angiogram (DCA) revealed bilateral indirect CCFs (type D on the right, type B on the left) with significantly higher flow on the right compared to the left (Figs 1C, 2A, 3A). A day later, through micro puncture of the right common femoral artery, coil was advanced (under real-time sonographic guidance and DCA). A microcatheter was used to access the right cavernous sinus via the left cavernous sinus through the intercavernous sinus. Right indirect CCF with arterial feeders arising from both the internal and external carotid arteries with outflow through the intercavernous sinus was confirmed. Embolization of the right cavernous sinus and intercavernous sinus was performed with 11 detachable coils and resolution of the type D carotid-cavernous flow was immediately noted (Fig. 2B); spontaneous resolution of the left type B CCF was noted (Fig. 3B) over a 24-h period of time.

Over the course of 2 months, the patient’s vision improved to 20/20 in the right eye with normalization of IOP to 11 mmHg and recovery of extraocular movements. Proptosis and signs of conjunctival vascular congestion completely resolved in a week (Fig. 1B).

## DISCUSSION

Although various classifications of CCFs have been cited in the literature, reports of bilateral, spontaneous, combined Barrow type D and B type CCFs remain limited. We present the case of an older woman without history of head trauma, albeit with a remote history of meningioma resection, presenting with a unique variant of bilateral indirect CCFs successfully treated with unilateral endovascular therapy.

Ophthalmic complications secondary to CCFs occur as a result of congestion from impaired venous drainage of the orbit to the cavernous sinus. Clinical presentation is variable due to differences in fistula size, location, rate of flow and location in the cavernous sinus [4]. Patients with direct CCFs classically present with the acute-onset clinical triad of chemosis (55–100%), proptosis (72%) and an ocular bruit (80%) [1, 4]. Other findings include eyelid edema, conjunctival arterialization, elevated IOP, ocular misalignment, diplopia and decrease in vision [1–3]. Diagnosis of CCF suspected clinically, is confirmed with contrast-enhanced CT angiography (CTA), MRA of the brain or diagnostic cerebral angiogram (the gold standard).

Indirect or dural, low-flow CCFs are uncommon clinical entities, typically occur spontaneously, and are insidious in onset. Bilateral, spontaneous indirect CCFs are rare, with a total of 35 reported cases in the literature [8]. There have been only two reported cases of bilateral CCFs involving a combined Barrow type D and B classification [9, 10]. Unlike the patient presented in this study, these patients either underwent a staged, bilateral-surgical arterial embolization or were managed conservatively with complete resolution of symptoms. Unlike direct CCFs, which are rarely asymptomatic and are treated urgently, indirect CCFs may resolve spontaneously due to thrombosis in up to 60% of cases [1, 4]. Over all, conventional endovascular therapy remains the mainstay of treatment. About 80% of patients who undergo endovascular repair experience resolution of CCF [4]. Interestingly, as seen in our patient, certain cases of bilateral CCFs may be sufficiently treated with unilateral endovascular treatment to obliterate both fistulas. About 20–60% of patients with indirect CCFs have spontaneous fistula closure [1].

Ocular involvement in CCFs may denote devastating vision-threatening complications with a delay in proper diagnosis and treatment. Venous congestion from raised venous pressure and resultant secondary glaucoma may disrupt ocular perfusion and result in irreversible vision loss. Both direct and indirect CCFs carry a favorable visual prognosis unless there is evidence of retinal or optic nerve ischemia prior to treatment [3]. Prompt diagnosis and treatment of CCFs can result in favorable visual outcomes. We therefore recommend considering the diagnosis of CCF when there is concern for possible orbital cellulitis, diplopia, an ICA aneurysm, or SOVT.

In summary, we describe a rare case of bilateral concurrent, non-traumatic indirect CCF (type B and type D confirmed on cerebral angiogram) successfully treated with endovascular coil embolization with spontaneous resolution of visual symptoms and immediate recovery of vision.
